# A one-step synthesis of rare-earth phosphate–borosilicate glass composites†

**DOI:** 10.1039/c8ra08657e

**Published:** 2018-11-20

**Authors:** Giovanni Donato, Derek Holzscherer, Jeremiah C. Beam, Andrew P. Grosvenor

**Affiliations:** Department of Chemistry, University of Saskatchewan Saskatoon S7N 5C9 SK Canada andrew.grosvenor@usask.ca

## Abstract

A new 1-step method for synthesizing glass–ceramic composites consisting of rare earth phosphates (REPO_4_) dispersed in borosilicate glass (BG) is reported herein as an alternative to the 2-step approach that is traditionally used. The effect of annealing time and annealing temperature on the formation of the 1-step glass–ceramic composites was investigated. Backscattered electron images and energy dispersive X-ray maps were collected to observe the morphology and chemical distribution in the glass–ceramic composites. X-ray diffraction was used to study the long-range order and X-ray absorption near edge spectroscopy was used to study the local environment of La, Y, P and Si. All analyses showed glass–ceramic composites made by the 1 and 2-step methods were similar to each other except for the Si L_2,3_-edge XANES spectra, which showed a slight change between the glass–ceramic composite materials made by the different synthesis methods. Xenotime-type phosphates (YPO_4_) were observed to be more soluble in the borosilicate glass than the monazite-type phosphates (LaPO_4_). This was attributed to the difference in the field strength of the rare-earth ions as a result of the difference in the ionic radii. Glass–ceramic composites made by the 1-step method were shown to form in 1 day at 1100 °C and in 3 days at 1000 °C without a significant change in glass or ceramic composition compared to the 1-step composite synthesized at 1100 °C for 3 days.

## Abstract 2

REPO_4_–BG composites synthesized by a new 1-step method were investigated and were found to be similar to the composite made by the traditional 2-step method.

## Graphical abstract

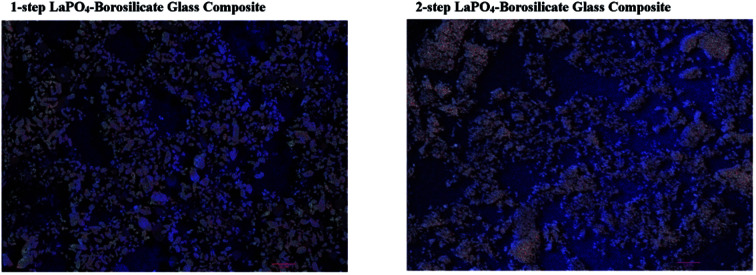

## 1 Introduction

Glass–ceramic composites are heterogeneous materials consisting of ceramic crystallites dispersed in a glass matrix. These materials are of interest because they can have improved chemical and mechanical properties compared to glass alone.^1,2^ The improved properties can be caused by the presence of crystallites in the glass and the properties can therefore be tailored to an application by changing the composition/amount of either the glass or ceramic phase.^3^ These materials have found uses for many applications, including: as photoluminescence materials; in fuel cells; as bioactive agents; as textiles; as integrated circuits; and as waste forms to sequester high level nuclear waste.^3–6^

High level nuclear waste is a product of the nuclear fuel cycle and consists, in part, of radioactive actinides.^7–9^ One method proposed to sequester high level waste is to incorporate these elements into a waste form followed by placement in a geological repository.^9–11^ This removes the radioisotopes from the environment and allows them to decay in a contained location. Three potential waste forms that have attracted attention are: glasses; ceramics; and glass–ceramic composites.^12–15^ Glass–ceramic composites capitalize on the advantages of both the glass and ceramic.^12,16^ The ceramic crystallites can incorporate actinides into the crystal structure giving the material a high waste loading potential as well as a high chemical resistance and the glass acts as a secondary barrier for the actinides while also being able to accommodate smaller fission products.^9,12,16^

**Fig. 1:**
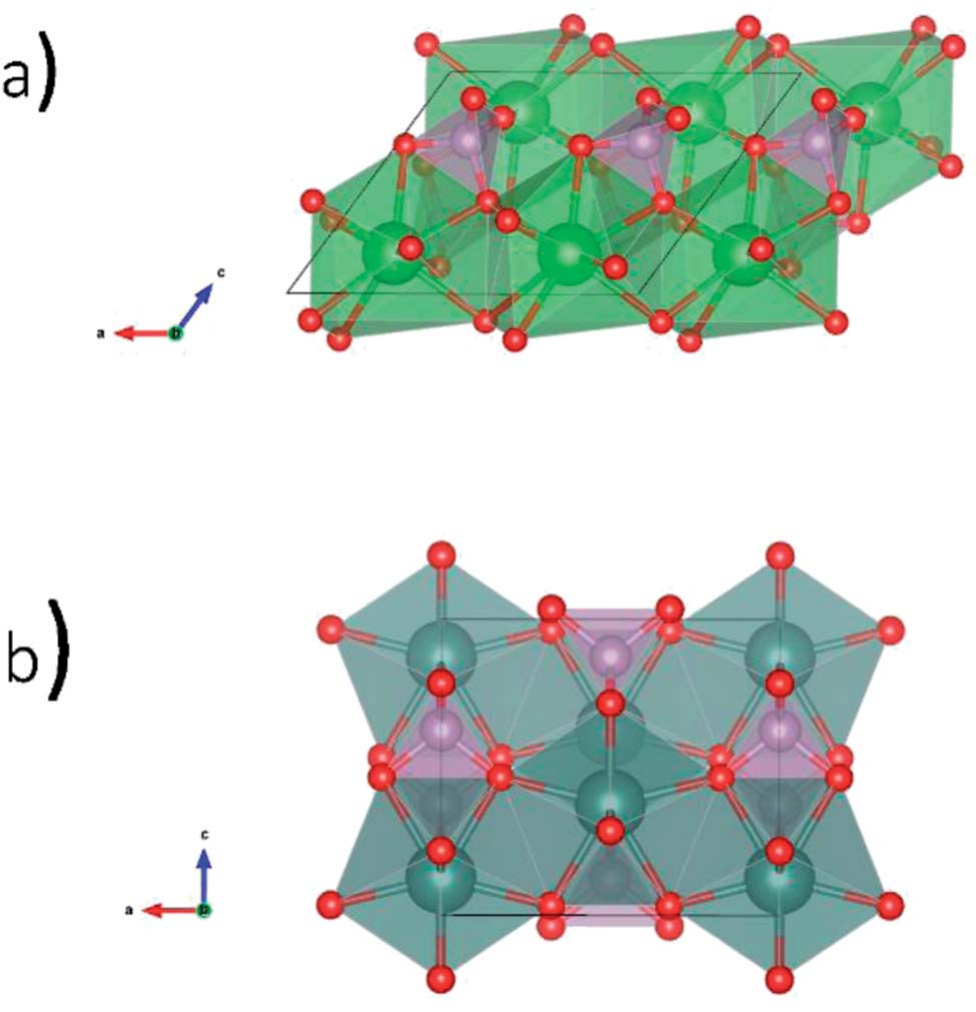
Crystal structures of REPO_4_ as (a) monazite (RE = La to Gd) and (b) xenotime (RE = Tb to Lu & Y).

Glass–ceramic composites containing rare-earth phosphates in a glass matrix have been proposed as a potential waste form to sequester high level nuclear waste.^2,17^ Borosilicate glass (BG) is one proposed type of glass to be used for this application because of the chemical durability of this material and the prospect of recycling glass from other industries.^18^ Natural rare-earth phosphates (REPO_4_) have been found to incorporate U and Th while remaining crystalline over geological timescales.^11,19^ Anhydrous rare-earth phosphates can adopt the monazite or xenotime structures.^20–22^ The monazite structure (Fig. 1a) is formed by the light rare-earth elements (La–Gd) and is monoclinic with the space group *P*2_1_/*n*.^13,19,23^ The rare earth ion in monazite is 9-coordinate while P is 4-coordinate. The xenotime structure (Fig. 1b) is formed by the heavy rare-earth elements (Dy–Lu, and Y) and is tetragonal with the space group *I*4_1_/*amd*.^13,19,24^ The rare earth ion in xenotime is 8-coordinate while P is also 4-coordinate in this structure.

Glass–ceramic composite materials are normally synthesized using a multistep process. One type of synthesis involves a 2-step method where the ceramic and glass phases are synthesized separately before being mixed and then annealed to form a composite material.^12,16,25–29^ The high level waste stream would be added during the production of the ceramic phase which would ensure all the minor actinides would be partitioned into the ceramic phase.^27^ A 1-step method is investigated here where all precursors are mixed together from the beginning followed by annealing. A 1-step method would have various advantages over a 2-step method. For example, a 1-step method would save on fabrication costs and be compatible with already existing glass producing infrastructure due to being able to form the glass–ceramic composite by the same melt-quench technique used to make glass.^30^ Additionally, the production of glass–ceramic composite waste forms by this method would be safer since the high-level waste would not need to be handled for as long a period. The partitioning of a complex waste stream into the ceramic and glass phases is outside the scope of this study; however, it is envisioned that the high-level waste stream would be incorporated during the single step reaction similar to the process used to make a glass waste form.^31^ Glass–ceramic composites containing LaPO_4_ or YPO_4_ crystallites dispersed in a borosilicate glass matrix were studied using powder X-ray diffraction (XRD), electron microprobe, and X-ray absorption near edge spectroscopy (XANES) to study the long-range order, morphology, and local chemical environment, respectively.

## 2 Experimental

### 2.1 Synthesis

**Scheme 1:**
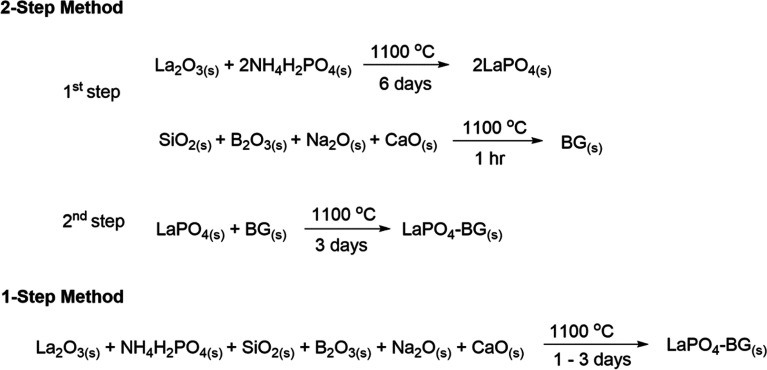
General synthesis strategies for making LaPO_4_–BG composites using 1- and 2-step synthesis methods.

The general synthesis strategies for synthesizing LaPO_4_–BG composites made by the 1- and 2-step methods are shown in Scheme 1.

#### 2.1.1 2-Step glass–ceramic composite synthesis

##### 2.1.1.1 REPO_4_

LaPO_4_ (monazite) was prepared using the ceramic method. La_2_O_3_ (Alfa Aesar; 99.9%) and NH_4_H_2_PO_4_ (Alfa Aesar; 99.9%) were ground and mixed stoichiometrically before being pressed into a pellet and annealed at 1100 °C for 6 days in an alumina crucible with grinding and pelleting occurring in 3 day intervals followed by quench cooling in air. In order to obtain pure phase xenotime, YPO_4_ (xenotime) was synthesized using a wet chemical method that was described previously by Kijkowska *et al.*^32^ Powdered Y_2_O_3_ (Alfa Aesar 99.9%) was added to 13.7 mL of 85 vol% H_3_PO_4_ and stirred to make a solution with a mole ratio of 1 : 100 (Y_2_O_3_ : PO_4_^3−^). The solution was then diluted by adding 100 mL of distilled water. After the water was added, the solution was refluxed at 130 °C for 2 hours and the precipitate was filtered and washed using distilled water. The precipitate was dried in a fumehood overnight before being pressed into a pellet and annealed at 1100 °C for 3 days in an alumina crucible followed by quench cooling in air.

##### 2.1.1.2 Borosilicate glass

Borosilicate glass was also made by the ceramic method. The mole percentage was as follows: 63.5% SiO_2_ (EMD Millipore), 16.9% B_2_O_3_ (Alfa Aesar; 99.98%), 16.5% Na_2_O (Alfa Aesar), and 3.1% CaO (Acros Organics; 97+%). The oxide powders were ground and mixed using a mortar and pestle, pressed into a pellet at 6 MPa, and annealed in 5% Au/95% Pt crucibles at 1100 °C for 1 hour followed by quench cooling in water.

##### 2.1.1.3 REPO_4_–BG composite

The 2-step glass–ceramic composites were formed by grinding and mixing the pure ceramic and glass with a 20, 40 or 50 wt% ceramic loading to make LaPO_4_–BG or YPO_4_–BG composite materials. The 20 and 40 wt% glass–ceramic composites were annealed in 5% Au/95% Pt crucibles at 1100 °C for 3 days followed by quench cooling in water. The 50 wt% ceramic LaPO_4_–BG composite made by the 2-step method was annealed for 1 hour at 1100 °C after grinding and mixing the pure ceramic and glass followed by quench cooing in water. The total synthesis time of REPO_4_–BG composites by the 2-step method was 6–9 days (6 and 3 days for the synthesis of LaPO_4_ and YPO_4,_ respectively, plus 3 days to form the composite). The glass–ceramic composite materials made by the 2-step method are described in Table 1.

**Table 1:** Sample names and compositions of the glass–ceramic composites studied

Sample name	Composition (RE = La, Y)	Synthesis method	Annealing time	Annealing temperature
20% 2-step	20 wt% ceramic REPO_4_–BG	2-Step	3 days	1100 °C
40% 2-step	40 wt% ceramic REPO_4_–BG	2-Step	3 days	1100 °C
50% 2-step	50 wt% ceramic REPO_4_–BG	2-Step	1 hour	1100 °C
20% 1-step	20 wt% ceramic REPO_4_–BG	1-Step	3 days	1100 °C
40% 1-step	40 wt% ceramic REPO_4_–BG	1-Step	3 days	1100 °C
50% 1-step	50 wt% ceramic REPO_4_–BG	1-Step	3 days	1100 °C
1100 C	40 wt% ceramic REPO_4_–BG	1-Step	3 days	1100 °C
1000 C	40 wt% ceramic REPO_4_–BG	1-Step	3 days	1000 °C
900 C	40 wt% ceramic REPO_4_–BG	1-Step	3 days	900 °C
3 days	40 wt% ceramic REPO_4_–BG	1-Step	3 days	1100 °C
2 days	40 wt% ceramic REPO_4_–BG	1-Step	2 days	1100 °C
1 day	40 wt% ceramic REPO_4_–BG	1-Step	1 day	1100 °C

#### 2.1.2 1-Step glass–ceramic composite synthesis

The 1-step glass–ceramic composite materials were synthesized using the ceramic method and the same precursors for the REPO_4_ and BG phases as for the 2-step synthesis method (see Section 2.1.1). The precursors were proportioned to give the glass–ceramic composite a theoretical ceramic loading of 20, 40, or 50 wt%. The stoichiometry of the glass forming and glass modifying oxides were the same as the pure glass made in the 2-step synthesis (mole percentage was 63.5% SiO_2_, 16.9% B_2_O_3_, 16.5% Na_2_O, 3.1% CaO). The reagents were ground, mixed and annealed in 5% Au/95% Pt crucibles for 1–3 days at annealing temperatures ranging from 900 to 1100 °C followed by quench cooling in water. The total synthesis time of the 1-step method was between 1–3 days. The glass–ceramic composite materials made by the 1-step method are also described in Table 1.

### 2.2 Powder X-ray diffraction

Powder X-ray diffraction patterns were collected using a PANalytical Empyrean instrument equipped with a Co or Cu Kα_1,2_ source or a Rigaku instrument equipped with a Cu Kα_1,2_ source. The patterns collected using the Co X-ray source were converted to the equivalent 2*θ* if a Cu source was used using the Bragg equation for consistency. Patterns were analyzed using the Powder Cell for Windows (PCW) software program.^33^

### 2.3 Electron microprobe

Electron microprobe images were collected from the 50 wt% ceramic LaPO_4_–BG composites synthesized by the 1 and 2-step methods as well as the 40 wt% ceramic YPO_4_–BG composites synthesized by the 1 and 2-step methods. Backscattered electron (BSE) images and electron dispersive X-ray spectroscopy (EDX) maps as well as EDX spectra were collected using a JEOL 8600 electron microprobe instrument. The samples were prepared by polishing the surface using diamond paste and were coated in carbon to reduce charging effects. The images and maps were collected using a magnification of 1000×.

### 2.4 XANES

#### 2.4.1 La L_1_- and Y K-edge XANES spectra

The La L_1_- and Y K-edge XANES spectra were collected using the Sector 20 bending magnet (20BM) beamline located at the Advanced Photon Source (APS), Argonne National Laboratory.^34^ The La L_1_-edge XANES spectra were collected from LaPO_4_ and the glass–ceramic composites synthesized by the 1 and 2-step methods having a 20, 40 and 50 wt% ceramic loading. The Y K-edge XANES spectra were collected from YPO_4_ and the 1- and 2-step glass–ceramic composites with ceramic loadings of 20 and 40 wt%. Samples were prepared by brushing finely ground powders on Kapton tape followed by folding the tape to produce multiple layers. The thickness was varied to maximize the absorption edge by adding or removing layers. Spectra were collected in transmission mode using a Si (111) double crystal monochromator, which provides a photon flux of ∼10^11^ photons per second. The spectral resolution of the La L_1_-edge XANES spectra was 0.6 eV at 6266 eV and the spectral resolution of the Y K-edge XANES spectra was 2.0 eV at 17 038 eV.^34^ The La L_1_-edge XANES spectra were collected using a step size of 0.15 eV through the absorption edge. The ratio of He : N_2_ in the ionization chambers during collection of the La L_1_-edge XANES spectra was 70% He : 30% N_2_ in the I_0_ chamber and 100% N_2_ in the I_t_ and I_ref_ chambers. The La L_1_-edge XANES spectra were calibrated by collecting a Cr K-edge XANES spectrum from Cr metal foil and setting the first derivative of the absorption edge to 5989 eV.^35^ The Y K-edge XANES spectra were collected using a step size of 0.3 eV through the absorption edge. The ratio of Ar : N_2_ in the ionization chambers during the collection of the Y K-edge spectra was 100% N_2_ in I_0_ and 50% Ar : 50% N_2_ in I_t_ and I_ref_. The Y K-edge XANES spectra were calibrated by collecting a Zr K-edge XANES spectrum from Zr metal foil and setting the first derivative of the absorption edge to 17 998 eV.^35^ All XANES spectra were normalized, calibrated, and analysed using the Athena software program.^36^

#### 2.4.2 P and Si L_2,3_-edge XANES spectra

The P and Si L_2,3_-edge XANES spectra were collected using the Variable Line Spacing-Plane Grating Monochromator (VLS-PGM) beamline located at the Canadian Light Source (CLS).^37^ The P and Si L_2,3_-edge XANES spectra were collected from LaPO_4_–BG and YPO_4_–BG composite materials containing 20 and 40 wt% ceramic and synthesized using either the 1-step or 2-step method. P L_2,3_-edge XANES spectra were also collected from the pure LaPO_4_ and YPO_4_ ceramics while Si L_2,3_-edge XANES spectra were collected from the pure BG material. Samples were prepared by brushing finely ground powder on carbon tape. Spectra were measured under ultra-high vacuum in total fluorescence yield (TFY) mode using a step size of 0.05 eV through the absorption edge. The high energy grating monochromator was used to collect both spectra. The spectral resolution of the P L_2,3_- and Si L_2,3_-edge XANES spectra is 0.01 eV.^37^ The P L_2,3_-edge spectra were calibrated by collecting a P L_2,3_-edge spectrum from red phosphorous and setting the first derivative of the absorption edge to 130 eV.^35^ The Si L_2,3_-edge spectra were calibrated by collecting a Si L_2,3_-edge spectrum from elemental Si powder and setting the first derivative of the absorption edge to 100 eV.^35^

## 3 Results and discussion

### 3.1 Powder XRD

#### 3.1.1 LaPO_4_–BG composites

**Fig. 2:**
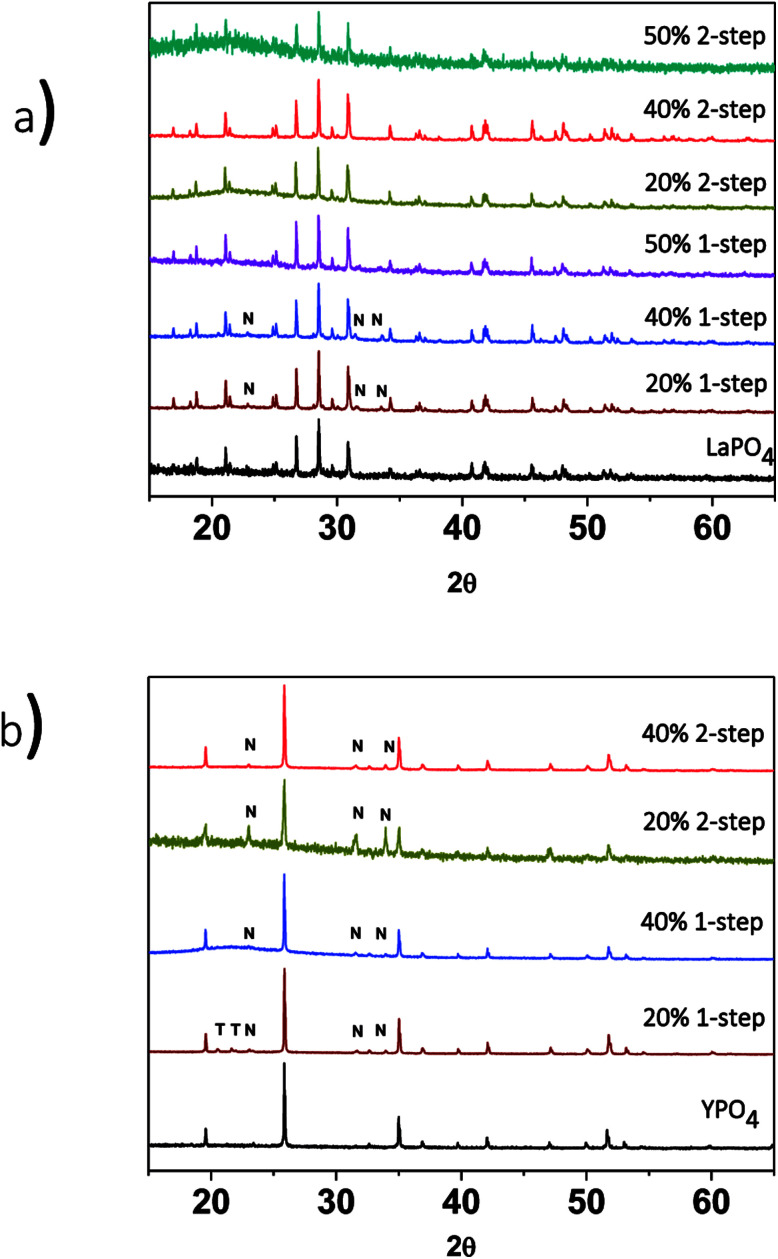
XRD patterns from (a) 1 and 2-step LaPO_4_–BG composites with 20, 40 and 50 wt% ceramic and (b) 1- and 2-step YPO_4_–BG composites with 20 and 40 wt% ceramic. T = tridymite peaks, N = nagelschmidtite phase peaks.

Powder XRD was used to characterize any crystalline phases present in the glass–ceramic composites made by the 1- or 2-step method. These materials consist of a glass matrix and ceramic crystallites; as such some powder XRD patterns were observed to contain broad humps representative of the glass phase in the glass–ceramic composite materials. The XRD patterns from the LaPO_4_–BG composites containing a 20, 40 or 50 wt% ceramic loading and made by the 1- and 2-step methods followed by annealing at 1100 °C are presented in Fig. 2a. The major crystal phase present in all LaPO_4_–BG composites was LaPO_4_.^38^ This demonstrates that in the 1-step synthesis, La^3+^ and PO_4_^3−^ formed a ceramic phase (at least primarily) instead of being incorporated into the glass matrix. Nagelschmidtite (Ca_7−*x*_Na_*x*_(PO_4_)_2+*x*_(SiO_4_)_2−*x*_) was also observed to be present in the XRD patterns from the 20 and 40 wt% ceramic composites that were synthesized using the 1-step synthesis method and was caused by the glass partially crystallizing. Nagelschmidtite, however, was not observed in the XRD pattern from the 50 wt% LaPO_4_–BG composite made by the 1-step method, which is likely a result of the high ceramic loading of this material and the expected low concentration of nagelschmidtite.

**Fig. 3:**
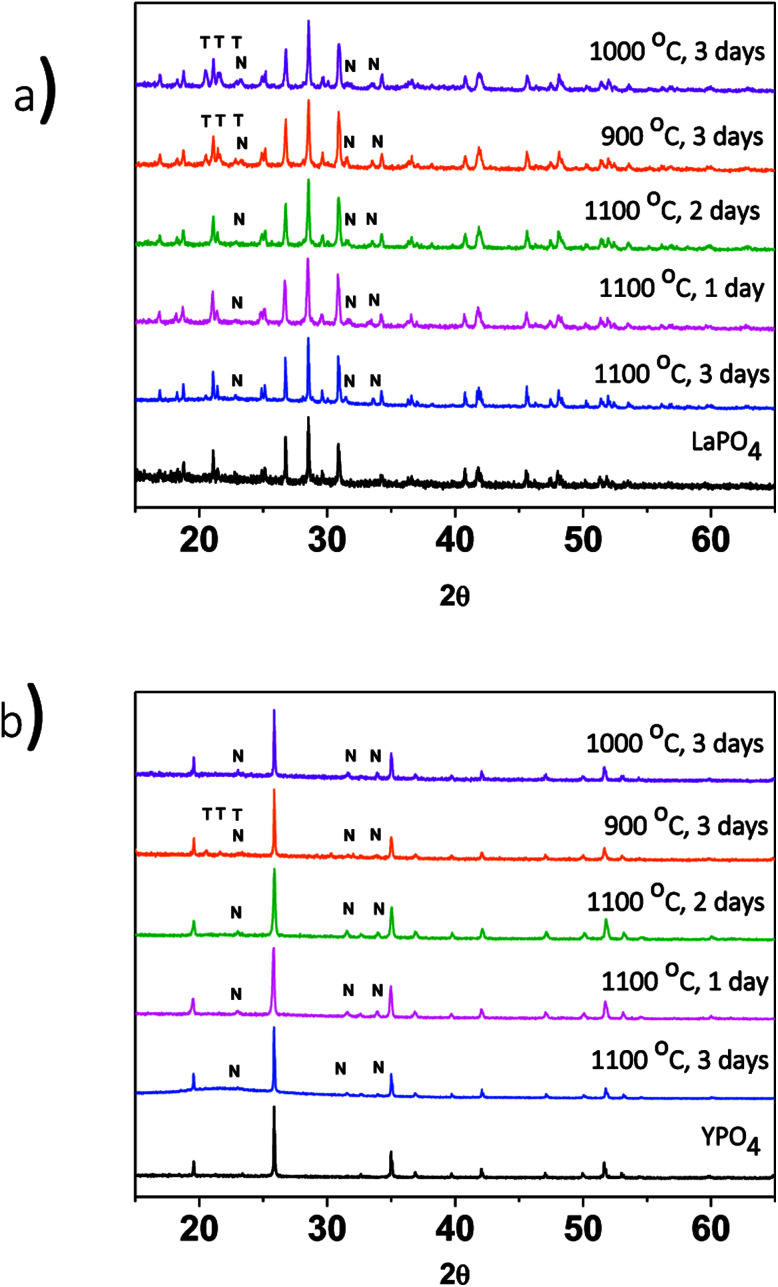
XRD patterns from (a) 1-step LaPO_4_–BG composites annealed at 900–1100 °C and 1–3 days and (b) 1-step YPO_4_–BG composites annealed at 900–1100 °C and 1–3 days. T = tridymite peaks, N = nagelschmidtite peaks.

The effect of changing the annealing temperature and annealing time on the crystal structure of the ceramic phase(s) was also investigated. The composites made by the 1-step method were synthesized at 1100 °C for 1, 2 and 3 days to observe how the structure changed depending on the annealing time. Fig. 3a shows the XRD patterns from the 40% LaPO_4_–BG composites annealed for 1, 2, and 3 days. Annealing for 1, 2, and 3 days at 1100 °C forms a composite which contains LaPO_4_ as the major phase and nagelschmidtite as the minor phase. An annealing time of one day is a much shorter time period to form monazite when compared to the 6 days it typically takes to form the pure ceramic at this temperature using the ceramic method.^13^ The decrease in time can be attributed to the glass being in a liquid phase at this temperature.^3^ This increases the mobility of the RE^3+^ and PO_4_^3−^ ions and allows them to diffuse through the sample and form monazite in a much shorter time.

The effect of annealing temperature on the formation of the glass–ceramic composites was also investigated by synthesizing glass–ceramic composites using annealing temperatures of 1100, 1000, and 900 °C for 3 days. Below 1100 °C, LaPO_4_, tridymite (crystalline SiO_2_), and nagelschmidtite were observed to form (Fig. 3a). The presence of crystalline SiO_2_ is unwanted in a waste form and should be avoided because crystalline SiO_2_ can undergo phase transformations at low temperatures, which leads to volume changes and the possibility of the waste form cracking.^29,39^ No changes in the 2*θ* positions of the diffraction peaks or changes in the intensity ratios of the diffraction peaks were observed, which suggests that little to no glass precursors were incorporated in the ceramic phase.

#### 3.1.2 YPO_4_–BG composites

XRD patterns from the 1 and 2-step YPO_4_–BG composites containing 20 and 40 wt% ceramic showed that the major crystal phase was YPO_4_ (Fig. 2b).^40^ Nagelschmidtite was also observed to form. The XRD pattern from the 40 wt% YPO_4_–BG composite made by the 1-step method also contained a very small amount of tridymite. Fig. 3b shows XRD patterns of YPO_4_–BG glass–ceramic composites annealed at 1100 °C for 1, 2, and 3 days. YPO_4_ was observed to form after annealing for only one day along with nagelschmidtite. Lowering the annealing temperature (1100–900 °C) when synthesizing the YPO_4_–BG composites (Fig. 3b) resulted in the formation of tridymite at 900 °C but not at 1000 or 1100 °C. Tridymite can form when glass–ceramic composites are annealed below the melting point of crystalline SiO_2_, which is what was observed when the annealing temperature was lowered when synthesizing both the LaPO_4_–BG and YPO_4_–BG composite materials.^41^ Once again there were no changes in the 2*θ* positions of the diffraction peaks or changes in the intensity ratios of the diffraction peaks observed suggesting that little to no glass precursors were incorporated in the ceramic phase.

### 3.2 Electron microprobe

#### 3.2.1 LaPO_4_–BG composites

**Fig. 4:**
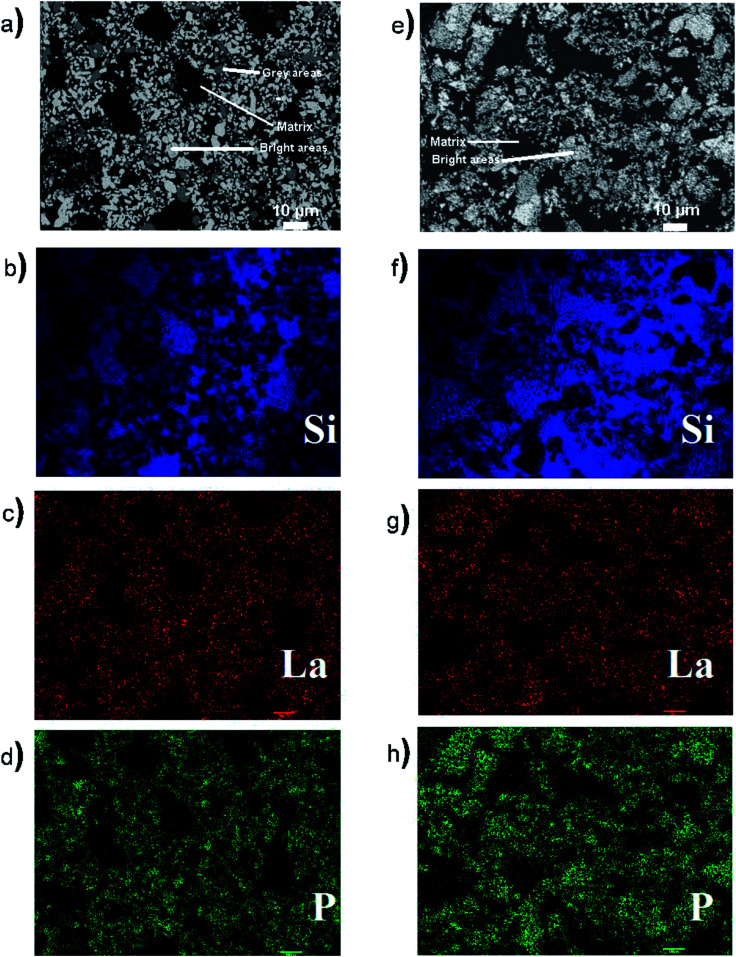
(a) Backscattered electron image of 50 wt% ceramic LaPO_4_–BG composite synthesized by the 1-step method, (b) Si EDX map of the composites made by the 1-step method, (c) La EDX map of the composites made by the 1-step method (d) P EDX map of the composites made by the 1-step method, (e) backscattered electron image of the 50 wt% ceramic LaPO_4_–BG composites synthesized by the 2-step method, (f) Si EDX map of composite made by the 2-step method, (g) La EDX map of the composite made by the 2-step method (h) P EDX map of the composite made by the 2-step method.

Backscattered electron (BSE) microprobe images were collected from the 50 wt% ceramic LaPO_4_–BG 1- and 2-step glass–ceramic composites (Fig. 4a and e, respectively). Two distinct regions can be observed in the micrographs of the glass–ceramic composites made by the 1- and 2-step methods: a dark matrix that represents the borosilicate glass and bright areas that represent the LaPO_4_ crystallites. There is an additional grey area in the micrograph from the glass–ceramic composite made by the 1-step method which may represent nagelschmidtite; however, this phase was not observed in the XRD pattern from the 50% LaPO_4_–BG composite made by the 1-step method (Fig. 2a). The crystallites were observed to be dispersed in the glass matrix in both glass–ceramic composites (*i.e.*, 1-step *vs.* 2-step). A difference in crystallite size and shape between the 1- and 2-step glass–ceramic composites was observed, which can be explained by how the crystallites formed in the glass–ceramic composites. In the 1-step method, the crystallites are formed in the glass while in the 2-step method, the pure ceramic is already formed and is mechanically ground before being mixed with the glass followed by annealing. In addition, the composite made by the 2-step method was only annealed for 1 hour which would not be expected to be sufficient for aggregation and crystallite growth to occur.^42^

Energy dispersive X-ray fluorescence (EDX) maps (Fig. 4) were also collected from 50 wt% LaPO_4_–BG composites annealed for 1 hour at 1100 °C to observe the elemental distribution in the different regions of the composite materials. The EDX maps from the composite material made by the 1-step method showed distinct regions containing high concentrations of P and La that correspond with the bright spots observed in the BSE image (Fig. 4d and c, respectively) while the matrix present in the BSE images showed a high concentration of Si in the corresponding EDX map (Fig. 4b), which corresponds to borosilicate glass. The EDX maps show discrete regions containing Si or La/P, respectively, indicating that there was very little, if any, La and P dissolved in the glass matrix. The EDX maps from the composite material made by the 2-step method also showed discrete regions containing La/P or Si (Fig. 4). The La and P EDX maps correlated with each other and correspond to the bright spots in the BSE image (*cf.*, Fig. 4e, g, and h). The Si EDX map correspond to the matrix in the BSE image (*cf.*, Fig. 4e and f). These results are similar to the results from the composite made by the 1-step method and further shows the similarity in the 1- and 2-step glass–ceramic composites despite the different synthesis methods.

#### 3.2.2 YPO_4_–BG composites

**Fig. 5:**
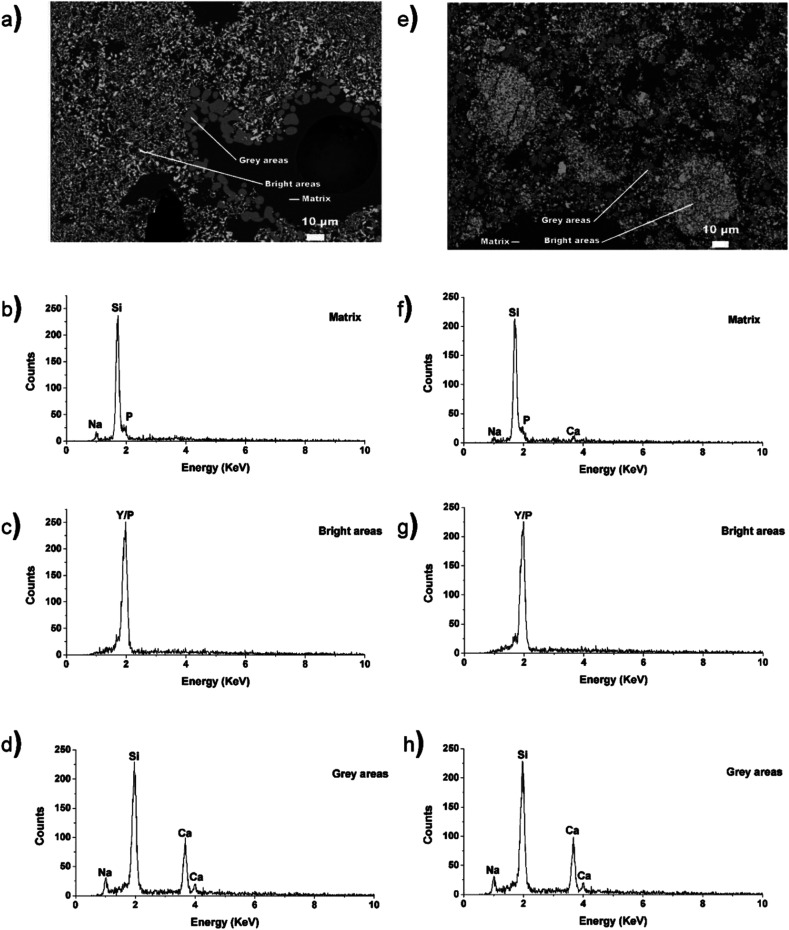
(a) Backscattered electron image of the 40 wt% ceramic YPO_4_–BG composites synthesized by the 1-step method, (b) EDX spectra taken of the matrix in the composite made by the 1-step method, (c) EDX spectra taken of the bright particles (YPO_4_) in the composite made by the 1-step method, (d) EDX spectra taken of the grey particles (minor crystal phase) in the composite made by the 1-step method, (e) backscattered electron image of the 40 wt% ceramic YPO_4_–BG composites synthesized by the 2-step method, (f) EDX spectra taken of the matrix in the composite made by the 2-step method, (g) EDX spectra taken of the bright particles (YPO_4_) in the composite made by the 2-step method, (h) EDX spectra taken of the grey particles (minor crystal phase) in the composite made by the 2-step method.

Fig. 5a and e show BSE images from the 40 wt% YPO_4_–BG composite materials made by the 1- and 2-step methods, respectively. These images were similar to the LaPO_4_–BG images in that there are crystallites dispersed in a glass matrix and that the shape and size of the crystallites was observed to change depending on if the 1- or 2-step synthesis method was used. Three regions were observed in the micrographs: a dark matrix, grey areas, and bright areas. The matrix represents the borosilicate glass, the grey areas are likely nagelschmidtite, while the bright areas are YPO_4_. The YPO_4_ crystallites were observed to be much smaller in these composites than the LaPO_4_ crystallites in the LaPO_4_–BG composite materials. This is likely due to YPO_4_ being more soluble in the glass than LaPO_4_. The difference in solubility between YPO_4_ and LaPO_4_ is discussed below when the Y K-edge XANES spectra are presented (Section 3.3.2). The micrograph of the YPO_4_–BG composite made by the 1-step method also shows a pore indicating the presence of air bubbles in the sample.

EDX spectra were collected from the different regions of the micrographs corresponding to the glass matrix, YPO_4_, and nagelschmidtite in the composite materials made by the 1- and 2-step synthesis methods. The spectra taken from the YPO_4_ crystallites in the composite materials made by the 1- and 2-step method (bright areas; Fig. 5c and g) indicate the presence of Y and P. Spectra taken from the grey areas present in the composites made by the 1- and 2-step method (Fig. 5d and h) indicate the presence of Si, Na, and Ca. The absence of P in the spectra is surprising given the general formula of nagelschmidtite, Ca_7−*x*_Na_*x*_(PO_4_)_2+*x*_(SiO_4_)_2−*x*_.^43^ Rare-earth elements (*e.g.* Nd) have also been found to stabilize the nagelschmidtite structure with higher silicon concentrations giving the general formula Ca_7−*x*_Nd_*x*_(PO_4_)_2−*x*_(SiO_4_)_2+*x*_ (0 ≤ *x* ≤ 1).^44^ A low intensity P signal in the EDX spectra could be overlapped by the large Si signal due to the proximity of the peaks and the resolution of the EDX detector used. The EDX spectra from the borosilicate glass (matrix; Fig. 5b and f) indicate the presence of Si, Na, P (and Ca in the case of the composite material synthesized by the 2-step method). This indicates that only a small amount of Ca was not crystallize into nagelschmidtite in the YPO_4_–BG composite material made by the 1-step method. The fluorescence signal from Y could be overlapped by the signal from P and Si in the EDX spectrum from the matrix (Fig. 5b and f) due to the similarity in energy of the peaks and the resolution of the detector. SEM and EDX maps were also collected from the 40 wt% YPO_4_–BG composite materials made by the 1- and 2-step methods from different spots on the samples (Fig. S1 in the ESI†). These maps suggest the presence of P and Y in the nagelschmidtite crystallite as well as the dissolution of YPO_4_ in the glass matrix.

### 3.3 XANES

#### 3.3.1 La L_1_-edge XANES spectra

**Fig. 6:**
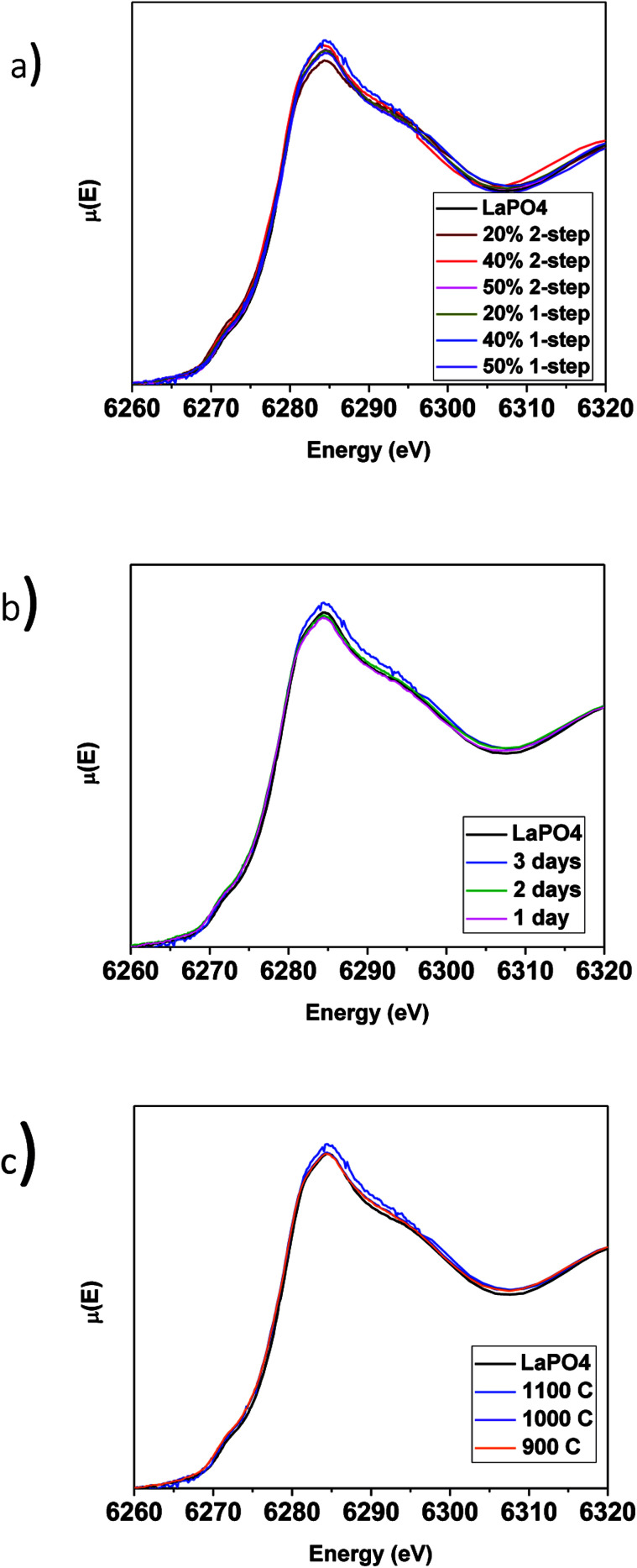
(a) La L_1_-edge XANES spectra taken from 1 and 2-step LaPO_4_–BG composites with 20, 40 and 50 wt% ceramic as well as pure LaPO_4_, La L_1_-edge XANES spectra taken from 40 wt% ceramic 1-step LaPO_4_–BG composites annealed at (b) 1–3 days at 1100 °C and (c) 900–1100 °C for 3 days.

The local chemical environments of the rare-earth elements in the LaPO_4_–BG composites were examined and compared to pure LaPO_4_ by collecting La L_1_-edge XANES spectra (Fig. 6). The La L_1_-edge XANES spectrum has been shown to consist of 2 features, a pre-edge feature and a main-edge feature. The pre-edge feature occurs due to a quadrupole, 2s → 5d transition while the main-edge feature is caused by a dipolar, 2s → 6p transition.^45^Fig. 6a shows the La L_1_-edge XANES spectra for the pure ceramic and the glass–ceramic composites with 20, 40, and 50 wt% ceramic made by the 1- and 2-step synthesis methods. The spectra from the glass–ceramic composites overlap with the spectrum of the pure LaPO_4_ ceramic, indicating that La in the glass–ceramic composites is in a similar chemical environment as La found in the pure-phase ceramic. This observation is consistent with the EDX maps from these materials (Fig. 4). The spectrum from the 20 wt% ceramic 2-step glass–ceramic composite showed slight changes when compared to the rest of the spectra. These changes in the La L_1_-edge XANES spectrum are likely caused by data collection/normalization issues since there is a low concentration of La in this particular sample and that the glass–ceramic composites made by the 1 and 2-step methods have been observed to be very similar when comparing the results from the other characterization techniques used in this study.

The La L_1_-edge XANES spectra from the glass–ceramic composite materials annealed for 1–3 days at 1100 °C and 1100–900 °C for 3 days are presented in Fig. 6b and c, respectively. All of the spectra were observed to be identical to the spectrum from the pure LaPO_4_ ceramic. Comparison of the La L_1_-edge XANES spectra (Fig. 6) and the XRD patterns (Fig. 2a and 3a) indicate that monazite can form within the composite materials using lower annealing temperatures than are generally used to form pure LaPO_4_ by the ceramic method and can form in as little as 1 day with little to no impact to the long-range order or local chemical environment of the LaPO_4_ monazite structure.

#### 3.3.2 Y K-edge XANES spectra

**Fig. 7:**
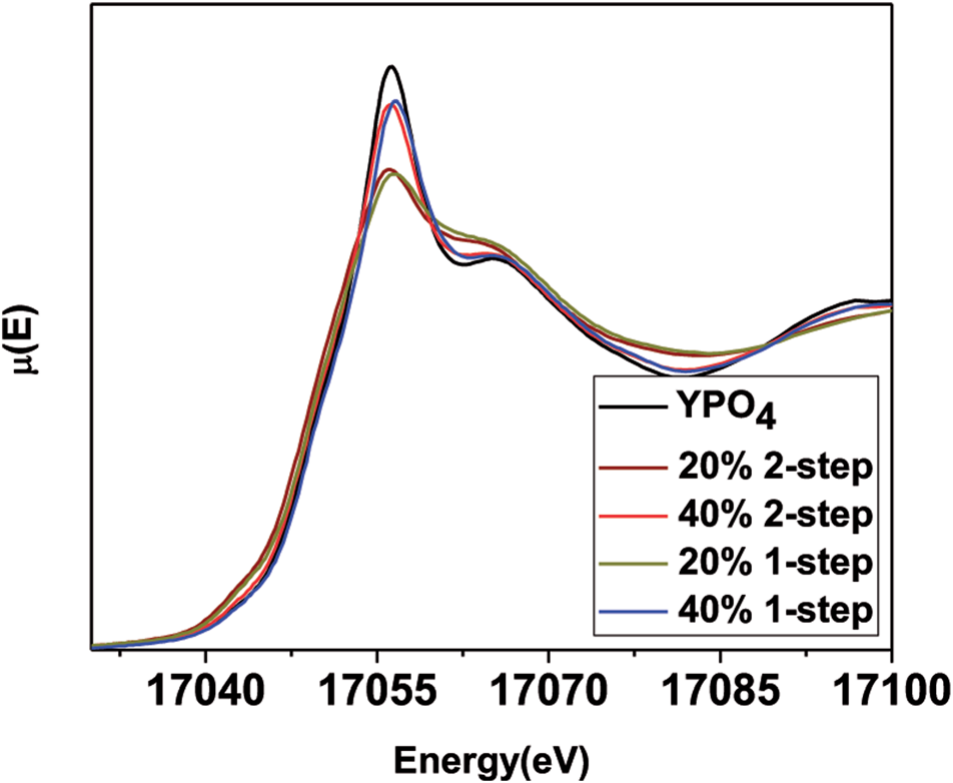
Y K-edge XANES spectra taken from 1 and 2-step YPO_4_–BG composites with 20 and 40 wt% ceramic as well as pure YPO_4_.

The local chemical environment of YPO_4_–BG composites was analyzed by collecting Y K-edge XANES spectra (Fig. 7). A Y K-edge XANES spectrum consists of a pre-edge feature and a main-edge feature. The pre-edge is due to quadrupolar, 1s → 4d transition while the main edge is a result of a dipolar, 1s → 5p transition.^46^ An increase in intensity of the pre-edge along with a decrease in intensity in the main edge is indicative of a decrease in the coordination number of Y.^47^ Overlap of the 4d and 5p orbitals increases when the coordination number decreases, which increases the dipolar character of the pre-edge transition.^48–50^Fig. 7 shows the Y K-edge XANES spectra from the glass–ceramic composites having a 20 and 40 wt% ceramic loading and made by the 1 and 2-step synthesis methods and are compared to the spectrum from pure YPO_4_. Yttrium has been shown to have a lower coordination number when it is dissolved in a glass matrix than in the xenotime-type structure.^40,51^

The intensity of the main edge feature in the YPO_4_–BG composite materials (Fig. 7) increases while the pre-edge feature decreases in intensity as the ceramic loading increases. The Y K-edge XANES spectra were observed to overlap when comparing glass–ceramic composites formed by the 1 and 2-step methods having the same ceramic loading. This shows that while the intensities of the features in the XANES spectra (and as a result the local chemical environment of Y) are dependent on ceramic loading, they are independent of synthesis method. The Y K-edge XANES spectra from all glass–ceramic composite materials show changes when compared to the spectrum from YPO_4_, which indicates that Y adopted a lower average coordination number in these composite materials. This suggest that Y dissolved in the borosilicate glass under all conditions studied. The proportionate amount of Y dissolved in the glass is greater in the 20 wt% ceramic than in the 40 wt% ceramic, which indicates that YPO_4_ will only dissolve and be incorporated into the borosilicate glass up to a point, with the remaining Y being found in YPO_4_. More of the crystalline phase will therefore be observed when more YPO_4_ is loaded into the composite material.

The difference in solubility of the rare-earth phosphates in the glass matrix between the LaPO_4_–BG composites and the YPO_4_–BG composites can be explained by examining the structural roles and field strength of the rare earth elements. Field strength is based on the theory of coulombic fields and can help predict and explain how the different cations interact with each other in glass.^52^ This is done by looking at how the cations interact with and compete for O^2−^ in order to satisfy their own local chemical requirements.^52^ The field strength is calculated by dividing the charge of the cation by the square of the cation–oxygen bond distance (FS = *z*/*r*^2^).^53–56^ Cations in a glass can be categorized as a network former, which makes up the glass network with bridging oxygen anions, a network modifier, which breaks up the network by introducing non-bridging oxygen anions, and network intermediates, which can act as either network formers or network modifiers depending on the glass composition.^57^ The category a cation falls into is largely based on the field strength and the bond strength between the cation and oxygen.^10,56^ Network modifiers have a general range in field strength from 0.1 to 0.4 and a typical bond strength of 10–50 kcal mol^−1^ whereas network intermediates have a field strength range of 0.5 to 1.0 and a typical bond strength of 50–70 kcal mol^−1^ while network formers have a field strength of >1.0 and bond strengths of >70 kcal mol^−1^.^10,56^ The rare-earth ions La^3+^ and Y^3+^ have field strengths of 0.50 and 0.57, respectively, and bond strengths of 58 kcal mol^−1^ (La–O) and 50 kcal mol^−1^ (Y–O).^55,56,58^ Y^3+^ has a higher field strength (0.57) than La^3+^ (0.50) due to the smaller ionic radius of Y^3+^.^55^ La^3+^ is on the edge of the field strength range between network modifiers and network intermediates and can act as a network modifier.^55^ Y^3+^ has been shown to break up the glass network or strengthen it in silicate glasses depending on composition and therefore it has been suggested that Y^3+^ can be classified as a network intermediate.^51,59^ This was also suggested to be true by Malchukova *et al.* for Gd^3+^, which has a similar field strength to Y^3+^ (0.55).^18^ In that study, Gd was doped into aluminoborosilicate glass (0–4.4 wt% of Gd_2_O_3_) and exposed to radiation in order to determine how these conditions effects the potential waste form.^18^ Of the amount of Gd^3+^ that was homogenously dissolved in the glass, the ratio of Gd^3+^ ions that acted as a network former and network modifier remained the same from 0.9–4.4 wt% of Gd_2_O_3_.^18^

By having a higher field strength, Y^3+^ is able to compete for O^2−^ more successfully than La^3+^ and behave as a network intermediate, leading to a higher degree of incorporation into the glass structure and a higher solubility in borosilicate glass. It has also been shown that crystallization is more difficult to induce in rare-earth doped borosilicate glass when Y^3+^ is the rare earth compared to when La^3+^ is present, indicating a higher stability of Y in the glass matrix caused by the difference in structural roles.^51^

#### 3.3.3 P L_2,3_-edge XANES spectra

P L_2,3_-edge XANES spectra were collected to study how the local chemical environment of P changed in the composite materials compared to the pure-phase ceramics. The P L_2,3_-edge XANES spectra can be used to study next nearest neighbour effects in the crystallites due to distortions in the PO_4_ tetrahedra affecting the spectra.^24,60^ These effects can be observed due to the lifetime of the core-hole created after the excitation. The core-hole in this spectrum has a longer lifetime than the core-hole in the La L_1_- and Y K-edge XANES spectra, resulting in a higher resolution.^23^ The features observed in P L_2,3_-edge XANES spectra result from 2p → 3s and 2p → 3d excitations. The 3s and 3d orbitals overlap in energy producing complicated transitions and spectra containing many features.^23^

##### 3.3.3.1 LaPO_4_–BG composites

**Fig. 8:**
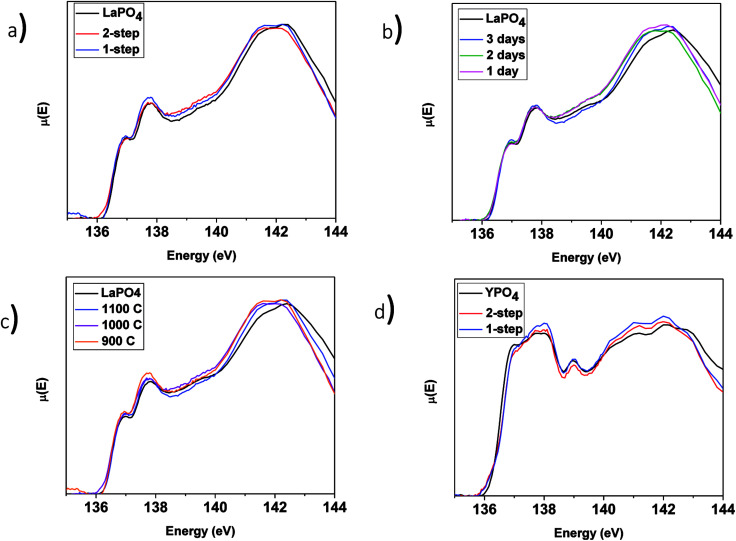
P L_2,3_-edge XANES spectra of (a) 1 and 2-step 40 wt% ceramic LaPO_4_–BG composites, 1-step LaPO_4_–BG composites annealed at (b) 900–1100 °C for 3 days, (c) 1–3 days at 1100 °C as well as pure LaPO_4_ and (d) 1 and 2-step YPO_4_–BG composites with 20 and 40 wt% ceramic as well as pure YPO_4_.

The P L_2,3_-edge XANES spectra from the LaPO_4_–BG composites and pure LaPO_4_ are shown in Fig. 8 and were collected to observe any changes in the local chemical environment of P in the composite materials *vs.* the pure-phase ceramic. The P L_2,3_-edge XANES spectra from all LaPO_4_–BG composites and LaPO_4_ were observed to overlap, which suggests no change in the local chemical environment of P regardless of synthesis method, annealing temperature, or annealing time used to form the composite materials. Comparison of the La L_1_-edge and P L_2,3_-edge XANES spectra indicate that the local environment of La and P remain the same in the composite materials compared to pure LaPO_4_. This being said, this suggestion could be questioned when the P L_2,3_-edge XANES results from the YPO_4_–BG composites are considered (*vide infra*).

##### 3.3.3.2 YPO_4_–BG composites

The P L_2,3_-edge XANES spectra from the YPO_4_–BG samples (Fig. 8d) indicate little to no change when compared to the spectrum from the pure-phase YPO_4_ ceramic. This is in contrast to the results from EDX spectra (Fig. 5) and Y K-edge XANES spectra (Fig. 7) from the YPO_4_–BG composite materials. Based on those results, some YPO_4_ was concluded to be dissolved in the glass matrix from the composite materials *vs.* the pure phase ceramic. The similarity between the P L_2,3_-edge XANES spectra despite whether P is located in the glass or ceramic phases in the composite materials suggests that the chemical environment of P is similar in the glass and ceramic phases. The examination of P L_2,3_-edge XANES spectra alone is therefore not appropriate for analysis of REPO_4_–BG composites and should be paired with other characterization techniques when analyzing REPO_4_–BG systems.

#### 3.3.4 Si L_2,3_-edge

Si L_2,3_-edge XANES spectra were collected to examine how the synthesis conditions can affect the local structure of the glass matrix. The Si L_2,3_-edge XANES spectrum is comprised of two main features. The lower energy feature results (primarily) from 2p → 3s transitions while the higher energy feature results (primarily) from 2p → 3d transitions.^61,62^ Crystallization of the glass and next nearest neighbour effects are among the changes that can be observed in this spectrum.^61^ When going from amorphous to crystalline SiO_2_, the spectrum becomes sharper and shifts to higher energy. This is because the long-range order in crystalline SiO_2_ causes the final states to be more degenerate.^12^ These spectra also become narrower when metal cations are present in the glass matrix.^61^ The narrowing of the spectra is caused by next nearest neighbour effects in the Si–O–M bond. The metal cation affects the bond length and covalency of the Si–O bond and will affect the spectrum as a result.^16^

##### 3.3.4.1 LaPO_4_–BG composites

**Fig. 9:**
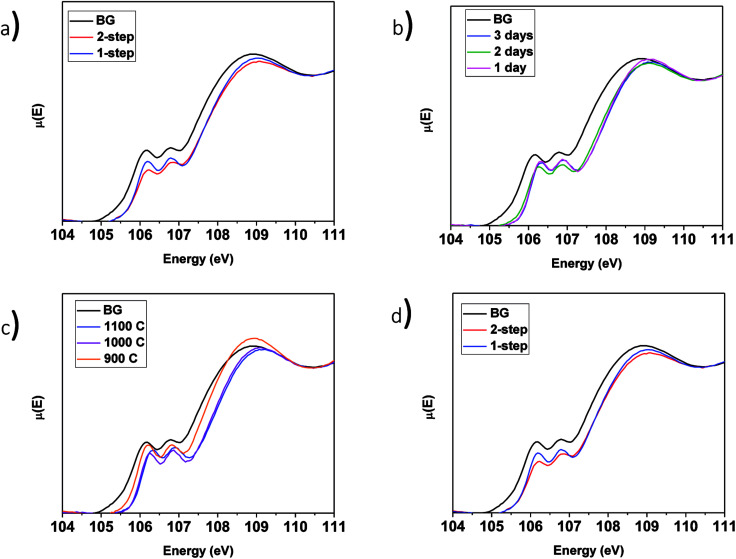
Si L_2,3_-edge XANES spectra of (a) 1 and 2-step 40 wt% ceramic LaPO_4_–BG composites, 1-step LaPO_4_–BG composites annealed at (b) 900–1100 °C for 3 days, (c) 1–3 days at 1100 °C and (d) 1 and 2-step YPO_4_–BG composites with 20 and 40 wt% ceramic as well as pure BG.

The Si L_2,3_-edge XANES spectra from the LaPO_4_–BG composites are presented in Fig. 9. Fig. 9a compares the spectra from LaPO_4_–BG composites synthesized using the 1- and 2-step methods. The intensity ratio of the L_2_ and L_3_-edges change depending on the synthesis method used. These changes have been attributed to changes in the ordering of the glass.^63^ The Si L_2,3_-edge XANES spectra presented in Fig. 9b overlap each other showing no significant changes in the local chemical environment of Si regardless of annealing time. The spectra presented in Fig. 9a and b from the glass–ceramic composites are narrower and higher in energy than the spectra from borosilicate glass. The changes in the spectra between the glass–ceramic composite materials and borosilicate glass are a result of next nearest neighbour effects caused by the presence of metal cations. Fig. 9c shows spectra from LaPO_4_–BG composites annealed at 1100, 1000, and 900 °C. The Si L_2,3_-edge XANES spectra from the composite materials become sharper with decreasing annealing temperature. The sharpening of the spectra is indictive of partial crystallization in the glass.^61^ XRD patterns from these glass–ceramic composites showed the presence of crystalline SiO_2_ when they were annealed for 3 days at 1000 and 900 °C (Fig. 3a). Analysis of the Si L_2,3_-edge XANES spectra suggest that the amount of crystallization that occurred at 1000 °C was minor and did not affect the overall structure of the amorphous glass matrix in a significant way while analysis of the spectrum from the composite annealed at 900 °C suggested that the crystallization of SiO_2_ impacted the Si network to a much greater extent.

##### 3.3.4.2 YPO_4_–BG composites

The Si L_2,3_-edge XANES spectra of YPO_4_–BG composites made by the 1 and 2-step synthesis methods are narrower to higher energy than the spectrum from pure borosilicate glass. These changes, like in LaPO_4_–BG composites, are a result of next nearest neighbour effects caused by the metal cation in the Si–O–M bond except in these cases it is from Si–O–Y. When compared to each other, YPO_4_–BG glass–ceramic composite materials synthesized by the 1- and 2-step methods show the same changes observed in the LaPO_4_–BG composites (Section 3.3.4.1) in that the intensity ratio of the L_2_ and L_3_ edges changed depending on the synthesis method. The cause of the change in the glass ordering in these materials seems to be linked to the synthesis method itself and not by how much ceramic dissolves in the glass. This is because the amount of ceramic dissolved in the glass was the same regardless of synthesis method and also this change in relative intensities was observed in the Si L_2,3_-edge XANES spectra from LaPO_4_–BG composite materials, where no dissolution took place.

## 4 Conclusions

Composite materials of rare-earth phosphate crystallites dispersed in borosilicate glass made by the 1- and 2-step methods were synthesized. Powder XRD, electron microprobe, and XANES have been utilized to examine the long-range order, morphology, chemical distribution, and local environment of glass–ceramic composites formed using either a 1- or 2-step method. Glass–ceramic composite materials made by the 1- and 2-step method showed either LaPO_4_ or YPO_4_ as the major crystal phase depending on the rare earth oxide used. All other characterization techniques showed 1- and 2-step composites containing the same rare-earth ion were similar to each other except by Si L_2,3_-edge XANES. The change in the relative intensities of the Si L_2_ and L_3_-edge are indicative of a change in the ordering in the glass as a result of the new synthesis method (*i.e.*, 1-step).

Glass–ceramic composite materials were also able to be synthesized in 1 day at 1100 °C and in 3 days at 1000 °C without any significant change in the glass or ceramic phases relative to the composite material synthesized for 3 days at 1100 °C. Different solubilities of LaPO_4_ and YPO_4_ in borosilicate glass were observed due to the different field strength of the rare earth ion present in the composite material. This difference in field strength lead to a difference in structural roles of La^3+^ (network modifier) and Y^3+^ (network intermediate). Ceramic crystallites dissolving in the glass matrix within a glass–ceramic composite waste form would minimize the inherent strengths of this class of waste form such as increased chemical stability and a double barrier (ceramic + glass). Consideration of the field strength of the ions used must be accounted for when synthesizing this system for nuclear waste sequestration applications. The 1-step synthesis method has shown potential as a synthetic strategy to prepare glass–ceramic nuclear waste forms.

## Conflicts of interest

There are no conflicts to declare.

## Supplementary Material

RA-008-C8RA08657E-s001
