# Supplementary material for: A one-step synthesis of rare-earth phosphate–borosilicate glass composites
Source: RSC Adv. 2018 Nov 20;8(68):39053–65. doi: 10.1039/c8ra08657e (PMC9090658; doi:10.1039/c8ra08657e)
Supplement: RA-008-C8RA08657E-s001 [file RA-008-C8RA08657E-s001.pdf]

Electronic Supplementary Material (ESI) for RSC Advances.
This journal is © The Royal Society of Chemistry 2018

Supporting Information

A One-Step Synthesis of Rare-Earth Phosphate-Borosilicate Glass Composites

Giovanni Donato, Derek Holzscherer, Jeremiah C. Beam, and Andrew P. Grosvenor*

Department of Chemistry, University of Saskatchewan, Saskatoon, SK, Canada, S7N 5C9

*Author to whom correspondence should be addressed

E-mail: andrew.grosvenor@usask.ca

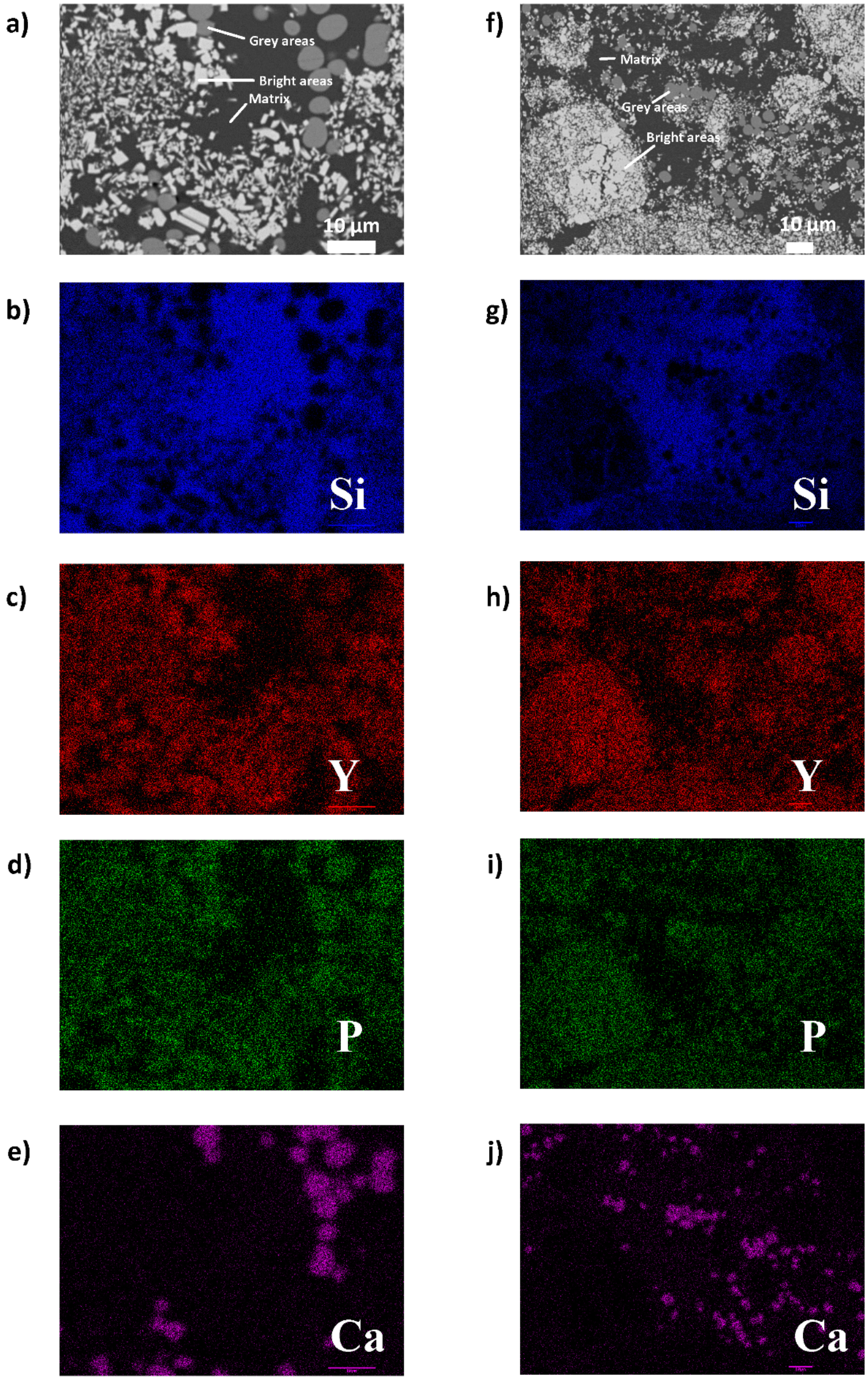
a)
Grey areas
Bright areas
Matrix
10 µm
b)
Si
c)
Y
d)
P
e)
Ca
f)
Matrix
Grey areas
Bright areas
10 µm
g)
Si
h)
Y
i)
P
j)
Ca

Figure S1: (a) Backscattered electron image of 40 wt% ceramic YPO_4-BG composites synthesized by the 1-step method, (b) Si EDX map of composites made by the 1-step method, (c) Y EDX map of composites made by the 1-step method (d) P EDX map of composites made by the 1-step method, (e) Ca EDX map of composites made by the 1-step method, (f) backscattered electron image of 40 wt% ceramic YPO_4-BG composites synthesized by the 2-step method, (g) Si EDX map of composites made by the 2-step method, (h) Y EDX map of composites made by the 2-step method (i) P EDX map of composites made by the 2-step method, (j) Ca EDX map of composites made by the 2-step method
